# Context, mechanisms and outcomes of a social enterprise model of residential rehabilitation for problem substance use: A realist‐informed process evaluation

**DOI:** 10.1111/add.70459

**Published:** 2026-04-29

**Authors:** Martin Anderson, Lucy Pickering, Mark McCann

**Affiliations:** ^1^ School of Health and Wellbeing University of Glasgow Glasgow UK; ^2^ School of Social and Political Sciences University of Glasgow Glasgow UK

**Keywords:** addiction recovery, drug‐related deaths, process evaluation, realist evaluation, recovery community, residential rehabilitation, river garden, substance use treatment

## Abstract

**Background and aims:**

This study evaluated a three‐year residential rehabilitation programme, which aimed to support recovery from problem substance use via peer support and social enterprise activities. The aims were to clarify programme mechanisms and identify contextual factors associated with variation in outcomes.

**Methods:**

The study took place within River Garden, a residential rehabilitation for problem substance use, based in South Ayrshire, Scotland. A mixed‐methods realist‐informed process evaluation was undertaken, using participant observation, repeated qualitative interviews and routinely collected admissions data. Fieldwork was conducted with residents, staff and trustees between April 2019 and November 2020. Nine (of ten) residents were recruited into the study. All residents were male, aged 20–47 years (median 35 years) and were White Scottish or English. Data collection and analysis was guided by Medical Research Council guidance on process evaluation and informed by selected principles from realist evaluation.

**Results:**

Three key contextual factors and six key mechanisms were associated with variation in resident outcomes. The severity of residents' substance use problems, their physical and mental health and their socioeconomic backgrounds shaped whether they responded to the programme's instrumental and relational resources with trust, respect or motivation (constituting six mechanisms, e.g. instrumental‐respect, relational‐trust). The programme was most beneficial for residents for whom intended outcomes were less constrained by contextual moderators.

**Conclusions:**

In residential rehabilitation for substance use disorders, residents with higher problem severity, worse physical and mental health and greater socioeconomic disadvantage appear to be less likely to respond to rehabilitation resources with trust, respect or motivation compared with the other residents. These findings may support the development of strategies to improve outcomes for residents with greater contextual barriers to change.

## INTRODUCTION

### The recovery movement in policy and theory

Recovery from problem substance use has been defined as a process involving the attainment of abstinence, health and citizenship [1]. It is a contested term, often measured in terms of improvement across a range of social, psychological, physical, financial and spiritual domains and not necessarily requiring abstinence [2]. The concept of recovery has gained prominence in international drug policy in recent years, particularly in the United States (US), Australia and the United Kingdom (UK) [3]. In Scotland, there is a notable grassroots recovery movement, including lived experience recovery organisations (LEROs), recovery communities and mutual aid fellowships, and the principles of recovery‐oriented systems of care (ROSC) have been well‐integrated into the national government strategies [4]. As Scotland experiences the highest rates of drug‐related death (DRD) in the United Kingdom, a national strategy was developed to tackle the issue via the expansion of both harm reduction and abstinence‐focused treatment infrastructure, integrating two distinct and often competing policy constellations [5].

Recovery‐oriented policy frames problem substance use as a consequence of a systemic breakdown of relationships within communities, meaning the solution is to imbue affected communities with the resources necessary to sustain recovery, creating organisations and institutions that facilitate community connections, housing and employment, health and wellbeing and positive identities and values [6]. Taken together, these constitute key components of ‘recovery capital’, the resources necessary to initiate and sustain recovery [7]. Recovery is also theorised to be closely tied to social identity change via the transformation of social relationships [8]. Critics agree on the importance of structural and systemic context, but argue that a policy‐level focus on abstinence risks creating increased harm [9] and influences the defunding of specialist harm reduction treatment services [10]. Furthermore, the focus on social relationships and identity has been critiqued as an example of responsibilisation, where responsibility for systemic problems is shifted from society to individuals [3, 9, 11]. That is, individuals experiencing systemic harms such as poverty and trauma are urged to manage risk by transforming their lives, while those most affected may have the least capacity to enact these transformations [3, 12]. Concerns have been raised that this approach will benefit communities with greater pre‐existing levels of the requisite forms of capital [13]. Study of policy constellations show that many actors recognise the dual value of community‐based provisions to increase recovery capital and system level change for enhanced harm reduction. At the same time, there is substantial polarisation between abstentionists who emphasise abstinence‐based residential rehabilitation and reformists who prioritise medication and overdose prevention, which has limited progress in enacting a cohesive policy response [5].

### Residential rehabilitation and the therapeutic community approach

Residential rehabilitation aims to support people to attain abstinent recovery, improved health and social functioning and enhanced quality of life [14]. Generally, this involves intensive psychosocial support, structured activities and is considered most suitable for people with significant physical, mental health or social problems [15]. Many residential rehabilitation programmes use the therapeutic community (TC) model [14, 16]. This is defined as a drug‐free environment where people live together and participate in structured activities, mutual aid and self‐help, where the community itself is viewed as the main facilitator of change in the individual, via peer support and staff guidance [17].

The evidence relating to the mechanisms and efficacy of these approaches is contested. Proponents highlight a consistent association between retention and outcomes as evidence of TCs as a gold standard treatment for people with the most severe problems [17]. However, reviews have found low rates of retention and completion, with no evidence of reduced mortality [16]. One review demonstrated that completion rates varied from 9% to 56%, and that 21% to 100% had relapsed by follow up, and 20% to 33% were back in treatment after 6 months to 6 years after leaving treatment [18]. The mainly observational research is limited in disentangling treatment effects from selection effects [16] and processes of natural recovery [19].

### The River Garden model

River Garden is a variation on the TC model, with a specific emphasis on the role of social enterprise activities and, notably, a lack of therapeutic intervention components. Meaningful work in high quality social enterprises was seen as key to successful recovery [20]. This was largely based on the San Patrignano recovery community in Italy [21], with the key difference that River Garden has semi‐permeable community boundaries to allow residents to pursue activities outside of the programme [20]. Structured work routines were intended to provide the context for interpersonal peer support, while increasing motivation, self‐esteem, responsibility and the vocational skills required to sustain recovery long‐term through employment [20, 21]. Originally launched in 2018, River Garden was a beneficiary in 2021 of £6 million to expand its capacity, as part of the Scottish Government National Mission to reduce DRDs [22].

### Study aims

Given the contested role of residential rehabilitation in the context of high DRDs [5], evaluation is required to better understand its role as part of a DRD reduction strategy and a framework of how it can be implemented. This study applies a process evaluation framework [23] incorporating principles from realist evaluation [24]. Specifically, we aimed to clarify the programme mechanisms and identify contextual factors associated with variation in outcomes [23].

## METHODS

### Study design

A mixed‐methods realist‐informed process evaluation was conducted. We conceptualised River Garden as a complex intervention, composed of multiple interacting components, flexibility in how it is delivered, targeting of difficult behaviours, interactions with its implementation context and potential for emergent outcomes. Process evaluation aims to understand implementation, mechanisms of change and how context affects outcomes [23, 25]. Specific principles from realist evaluation were used within this framework. First, mechanisms were conceptualised as cognitive or affective responses to specific programme resources [24, 26]. Second, we incorporated realist interviewing techniques to help sense‐check and refine our theoretical conclusions [27]. We did not attempt to consolidate full context‐mechanism‐outcome configurations or theorise generative causation.

### Setting and participants

Participants were recruited from the River Garden project, a 47‐acre work‐based residential rehabilitation community for men based in a Victorian walled garden site previously owned by an agricultural college. Residents could progress from volunteer (0–3 months) to trainee (3–14 months) to paid staff (15–36 months). Before becoming staff, they were not paid but received free accommodation and meals. Volunteers/trainees received free accommodation and meals but were not paid, whereas staff were paid with deductions for accommodation. Volunteers were expected to participate in basic work and attend local recovery meetings, although could not leave the site unsupervised. Trainees were expected to work in or begin to develop a specific social enterprise and could leave unsupervised for training, education or recovery activities. Staff supervised new volunteers. In practice, much of the work involved gardening or construction work to restore the site, as the social enterprises were in early development and provided limited employment or training opportunities.

All residents were invited to participate in the study and nine of 10 residents were recruited. All residents were male, 20 to 47 years old (median = 35 years). All were White Scottish or English. Five residents reported primary opioid use, often alongside benzodiazepines, cocaine and alcohol. Two reported their primary dependence was alcohol. One reported their primary drug was cocaine. One reported benzodiazepines, alcohol and novel psychoactive substances (NPS). All residents were unemployed. Five had spent time in prison (three more than once). Three reported poor physical fitness, while eight reported mental health issues (commonly anxiety/depression, but a minority mentioning paranoia, panic attacks, self‐harm, suicide attempts and psychiatric admissions). Ultimately, four residents were still residents at the end of fieldwork. Five had left the programme before completion.

Four staff members participated, constituting all full‐time staff members employed by the project during the research period. Four trustees were recruited, constituting a minority of all trustees involved in the project, as many trustees had left before this stage of the research. Two of these were ex‐trustees when interviewed.

### Data collection

Participant observation was conducted between April 2019 and March 2020. Interviews were conducted between April 2019 and Nov 2020. After March 2020, the coronavirus disease 2019 lockdown prevented further site visits and remaining interviews were conducted via videoconferencing software. Residents, staff and trustees participated in different procedures with some overlap.

#### Residents

Residents were the main focus of the participant observation, which involved the researcher participating in community activities. Fieldnotes were taken to record insights on peer mentoring, engagement with work, social support, identity, routines and relationships (Appendix S1). Each resident was interviewed at up to four time points: (1) within 3 months of arrival; (2) approximately 6 months residence; (3) approximately 12 months residence; and (4) a post‐exit interview if they left the community. At each time point, residents took part in a qualitative social network interview (Appendix S2), which involved a visual mapping exercise of their social contacts. The network map was used as visual prompt for residents to qualitatively discuss the structure, closeness and quality of their relationships [28, 29], which elicited insight into affective and cognitive mechanisms of social connection, as well as wider social context (e.g. ties to family or recovery communities). We also accessed resident admissions data for consenting participants, providing additional contextual information on substance use, health, family, criminal history *etc*.

#### Staff and trustees

Participant observation also involved interactions with staff and trustees. Early in the research, staff members took part in a single set of qualitative social network interviews. At the end of the research, a series of one‐off interviews were conducted with staff and trustees. This involved questions that were designed to help validate and refine our theorised mechanisms and contextual factors identified in earlier fieldwork [27] (Appendix S3).

### Ethical issues

Ethics approval was granted by the University of Glasgow College of Social Sciences Research Ethics Committee (Ref: 400180111). Amendments were approved permitting the use of remote research methods and to conduct the final staff/trustee interviews. A protocol was developed relating to any disclosure or observation of the risk of harm to oneself or others. Interviews were either scheduled in by staff members or directly arranged with residents during participant observation visits. Participants were provided with information sheets and consent forms, with separate consent options for the audio recorded interview, inclusion in participant observation and access to their admissions data. They were informed that participation was voluntary, non‐participation would not affect their status in the programme, they could withdraw at any time without giving a reason and request that any data already provided was not included in the study. Consent was recorded separately at each interview. One resident declined to take part, reassuring us that the optional and voluntary nature of the study had been clearly communicated. Another declined to inclusion in participant observation, so no fieldwork observations involving them were included. Participants were not paid for taking part, with the exception of a £20 payment for ex‐residents who were followed up. All participants are referred to by pseudonym.

### Analytical approach

Fieldnotes and interview transcripts were inductively coded using NVivo. We created high level codes for context, mechanisms and outcomes. Within these, we created subcodes for specific examples identified within the dataset. We defined contextual factors as anything external to the intervention that could affect how it works [23]. Mechanisms were defined as resource‐response interactions experienced by residents [24, 26]. Programme resources were broadly categorised into either instrumental or relational resources. This was based on research theorising that the two main forms of TC strategy are structured task completion and peer influence [30]. Resident responses were classed as cognitive or affective responses to these resources, with categories identified inductively from the transcripts and fieldnotes (Appendix S4). Outcomes were defined as observable behavioural events. Multiple rounds of coding led to a final set consisting of three contextual factors, six mechanisms and two outcomes (engagement/retention or disengagement/attrition). Initially, the lead researcher coded a random sample of transcripts to develop the initial coding framework, based on fieldwork with residents and staff. Co‐authors provided conceptual feedback to help refine the framework. This framework informed the topic guide for the final validation interviews with programme implementers, which were coded within the same framework.

### Positionality statement

The first author and fieldworker (M.A.) was a white cisgender male in his mid‐30s when the research was conducted. He was a PhD student at a Scottish higher education institution and is now employed as a postdoctoral researcher. Participant observation involved efforts to integrate into the community by joining in with resident activities, mainly in a specific 3‐month period, which involved regular visits and overnight stays in resident accommodation. He was particularly interested in how vocational and educational opportunities can provide recovery pathways and held a belief that recovery should be given greater emphasis in addiction treatment policy. Because of observation of divergent resident outcomes in this study, he developed a more balanced view that recovery‐oriented and harm‐reduction approaches may both be suitable for different people at different times. Although the entire team contributed to the manuscript and provided supervision throughout the project, the first author was primarily responsible for the conduct of the research.

## RESULTS

### Resident characteristics as a key contextual factor

Although all residents shared the characteristic of having experienced problem substance use, there was some variation in their backgrounds that led to divergent outcomes. Key areas of variation that directly affected the programme mechanisms were the problem severity of their substance use, their physical and mental health and their socio‐economic background. These factors tended to be interrelated and were often clustered within specific subgroups of residents.

#### Problem severity

The intake procedures required residents to be abstinent from illicit substances and substitute medications before entry, but there was substantial variation in the length of time in abstinence between residents. Two of the four residents who progressed positively and were still in residence when fieldwork concluded had over 6 months prior abstinence and viewed River Garden as an opportunity to rebuild their lives, with one saying he was ‘constantly just trying to figure out something I could do or for a job or where I could move to…all in I was six months clean and sober before I came in’ (Eric, resident, April 2019). There were also residents who entered following more recent drug use, one stating that during the application they were ‘probably still partly rattling [withdrawals]…you'd have signed anything to get away from that life’ (Kyle, resident, 2019). There was an understanding among staff that a higher level of stability increased the chances of positive outcomes and ‘people that are still chaotic are certainly…not for us’ (Trevor, staff, September 2020). This would include people ‘using lots of different kind of drugs, lots of overdoses’ (Neville, trustee, November 2020) or ‘people with…serious addiction, and really, near death's door…they're not set up for the complexity of problem that can come with that’ (Lindsay, trustee, November 2020).

#### Physical and mental health

Residents also varied in their physical and mental health on arrival. Residents with greater physical health could cope better with the physical demands of the structured work routines, which often involved outdoor gardening tasks or building work related to the renovation of the physical infrastructure of the site. Residents with better mental health could more effectively form relationships within the communal living environment. There were residents whose mental health improved because of the structure and purpose provided. However, physical and mental health problems could be exacerbated by the challenges of the programme, particularly as residents progressed and were challenged with greater responsibilities. An ex‐resident explained, ‘I've got arthritis in both my knees, both my wrists. I've got a bad back…my mental health was fucking getting bad, man, I couldn't do it anymore’ (Brian, ex‐resident, December 2019).

Those with severe mental health challenges often experienced mental health decline, particularly because of the challenges of receiving or providing peer support. One resident found that being challenged by peers made him angry, because ‘when you start getting paranoid, you become wary of everybody…people are challenging you…and people pushing your buttons, to the point you're ready to fucking explode’ (Stuart, resident, February 2020). Peer support could be ineffective for individuals who might require more specialist mental health support, because ‘we rely on the guys taking care of the guys…so, we can't put the person who would feel really ill…if they don't get the support’ (Nigel, staff, September 2020). Providing peer support could affect mental health, as one resident experienced that ‘addicts have got this uncanny ability of picking up on a weakness right away…they'll exploit it to the best of their ability’ (Brian, resident, April 2019). The large site meant many peer interactions occurred away from staff supervision. This restricted efforts to facilitate positive relationships, despite staff awareness of ‘all the nipping…verbal bullying’ (Trevor, staff, July 2019).

#### Socio‐economic background

There was a consistent theme that residents from higher socio‐economic backgrounds experienced more positive outcomes. All four of the residents who experienced successful outcomes noted this pattern, one commenting ‘I'd say I'm reasonably middle class…I've not struggled as much as other people…Whether it's nice to admit it or not, it's probably true’ (Paul, resident, February 2020). Often, the concept of socio‐economic status was used by residents, staff and trustees synonymously with the concept of having a supportive family background, with one resident observing ‘I come from a good family and stuff…Everybody that's still here now…has got a lot of family and support behind them compared to some of the people that have left’ (Eric, resident, December 2019). Staff also recognised that ‘anybody that we've had that has come from a…poorer background, haven't lasted’ (Trevor, staff, September 2020). Residents who had experienced high levels of criminalisation were considered particularly unsuitable, based on early experiences, that ‘jail behaviours are going to fucking come out of the jail and come straight in here’ (Keith, resident, December 2019). A newer resident was relieved to find that River Garden contained people of a similar socio‐economic background, as the website ‘was talking about people coming out of prison and whatnot, I had worries that it would be a bit rough’ (Neil, resident, April 2020).

### Relational and instrumental mechanisms

#### Relational mechanisms

Relational‐motivation relates to the motivation to engage in close interpersonal relationships. This included a motivation to provide and to receive peer support, to engage in external recovery communities and to interact with visitors to the project. A loss of peer‐related relational‐motivation could lead to residents leaving the community. For example, Brian excelled with the instrumental challenges, but began to struggle when progressing to a staff role as the cohort of new residents expanded and required supervision, becoming disheartened with the strain of close quarters living and minor domestic squabbles: ‘I couldn't go back into that accommodation block, now, with the boys…, sitting round at mealtimes with everybody… I don't want to do that again’ (Brian, ex‐resident, December 2019). However, it was also not a critical mechanism for residents who were retained. Although long‐term residents did have good relationships with each other, they led very independent, individualised vocational pathways, which did not require substantial peer support. A norm developed that independence rather than close peer supervision was more beneficial, because ‘it's quite important they have that downtime…when nobody is looking over their shoulder’ (Donny, staff, September 2020). However, this level of independence was less suitable for newer residents who may have benefited from close supervision, with one describing how he relapsed when ‘I was in conversation on the phone, and…things, kind of, got on top of me…an argument with the girlfriend…And I decided I wanted to go and get some cigarettes and…there was alcohol in the shop’ (Neil, ex‐resident, September 2020). Ultimately, relational‐motivation was hindered by the strong focus on structured work and training, meaning that established residents' capacity to provide meaningful peer support was diminished by the fact their time was largely taken up by individualised work‐focus activities.

Relational‐trust involved the capacity of residents to trust their peers and staff in the community. When this trust was present, residents could feel relaxed, able to be themselves and form the connections necessary to sustain a long‐term residence in the community. However, the capacity to trust was often impeded by contextual factors and the emphasis on independence, because of residents leading separate recovery pathways and engaging in broader social integration, led to limited efforts to build trust with new residents. Staff recognised there was a problem with new residents becoming isolated, but tended to frame this in terms of a problem with the attitude of the resident, for example ‘he [Stuart] didn't have the right attitude, he was definitely not a community member, he isolated all the time in his room’ (Nigel, staff, September 2020). However, the subject of these comments expressed this isolation in terms of a struggle to form trusting connections:

‘It is difficult to trust people in here, because I've been let down that many times in the past … when I retract to my own environment or room, I question closeness of people in here, I do. I don't know them well enough…when you let down your guard, you leave yourself open to fucking people taking the piss.’ (Stuart, resident, February 2020).

Relational‐respect involved interpersonal respect toward other community members. In the second year of implementation, a cohort of three new residents became disconnected from the initial cohort and generated a culture of low relational‐respect. This was based in perceived differences between themselves and the established residents, feeling more comfortable with each other, as well as feeling disrespected themselves by not receiving the vocational development opportunities they expected. Their behaviour made it difficult for staff to maintain order and led to a culture of verbal bullying toward established residents and staff, ‘it was just all flinging pelters at each other, all giving abuse’ (Kyle, ex‐resident, July 2019). In particular, they refused to respect attempts by long‐term residents to provide them with peer support, with one resident commenting that ‘they're quite challenging. They take the piss constantly, like to wind you up…try and get a reaction out of you all the time’ (Brian, resident, April 2019). The new residents worked collectively to resist and challenge integration into the community, supporting each other in arguments with staff, because ‘if you're close with somebody you back them up*’* (Kyle, ex‐resident, July 2019). This led to negative social influence, ‘we'd kind of made a pact, it was if Kevin got, like, kicked out, I was leaving, if I got kicked out, he was leaving’ (Kyle, ex‐resident, July 2019).

Relational mechanisms may have been achieved more effectively if the ‘resource’ element of the resource‐response had been implemented more effectively. One trustee felt that the programme implementation should have been more closely supported by existing recovery communities to establish a positive recovery culture, ‘you don't have enough grown‐up recovery, so you need to borrow it from outside’ (Lindsay, trustee, November 2020). With a more cohesive culture of supportive relationships, there were certain residents who likely could have experienced better outcomes, like Kyle who was ‘doing really well up until the point that these divisions came about’ (Trevor, staff, July 2019).

#### Instrumental mechanisms

Instrumental‐motivation was the critical programme mechanism. This involved enthusiastic engagement with opportunities for training and development. Although all residents noted that the work could be repetitive and unproductive, those residents highly motivated by the long‐term opportunities were able to view this as an opportunity to show initiative that would lead to more responsibilities. Over time, these residents developed specific social enterprise ideas: ‘I've now got a social enterprise that I kind of have started…as well as all the other day‐to‐day stuff’ (Paul, resident, February 2020). Staff recognised that allowing residents to build businesses related to their interests could increase motivation, because ‘they will take ownership of that’ (Nigel, staff, August 2019). However, other residents became demotivated when they felt meaningful training and development was not being offered quickly enough. This led to feelings of apathy and disengagement from the work routines, because ‘you just burn out after a wee while…you lose the will’ (Kyle, resident, April 2019). Voluntary work was felt by implementers to be a fair trade for access to rehabilitation and encouraged ‘gratitude for being alive…for being clean…for eating good food’ (Nick, trustee, November 2020). However, motivation diminished when residents felt work itself was not rehabilitative.

Instrumental‐trust was the level of trust residents had that the structures and routines of work were personally beneficial. Resident who progressed recognised that the work could provide them with a sense of purpose, because ‘not having that purpose drags you down…just doing all this stuff, work…you value yourself again’ (Paul, resident, August 2019). Furthermore, they were able to trust that demonstrating a good attitude would lead to more rewarding development opportunities at a later stage. Residents with low instrumental‐trust focused on the lack of more immediate rewards, ‘you're overworked in here. Sometimes you feel like you're in the…slaves or something like that’ (Kyle, resident, April 2019). Residents could lose belief in the value of work for resolving the underlying causes of substance use. One described it as ‘basically…a work camp for addicts’ (Brian, ex‐resident, December 2019), while another felt that work, in the absence of strong relational support, lacked therapeutic benefits and was a ‘distraction technique’ (Neil, ex‐resident, September 2020).

Instrumental‐respect was the extent to which resident respected the structures, routines and rules of the community. Relatively low levels of instrumental‐respect could be tolerated if residents were otherwise demonstrating instrumental‐motivation and relational‐respect. For example, the rules on smoking were not enforced strongly by residents or staff on site, who questioned ‘why focus on such a small thing like that’ (Ian, resident, December 2019). However, very low instrumental‐respect caused more serious problems in relation to more fundamental aspects of the programme. Fundamental aspects included routine, contribution and respecting boundaries, which were not always respected and needed to be managed ‘strictly’ by staff:

‘We need to be strict with, you know, you get up in the morning, you go to breakfast, you do this, you work, you don't have your phone, you don't smuggle phones in, you use your phone not on work times, you honour your commitments, if you're on cooking rota you don't just skip it and go outside…you're here to do your job’ (Nigel, staff, August 2019).

Instrumental mechanisms may have been achieved more effectively if the programme instrumental resources had been more fully developed before accepting residents. Staff recognised the importance of residents seeing ‘worth in what they're doing rather than just moving stuff about the place, or painting walls for the sake of it’ (Trevor, staff, September 2020). It was felt providing meaningful and motivating work would be easier ‘[when] we've got five, six or seven social enterprises that people can slot into…we might be able to take people with…other issues’ (Donny, staff, September 2020).

### Outcomes

All four residents who remained at the end of the study period attained responsibility for a specific vocational pathway: café, events, woodworking and grounds keeping. They also pursued development opportunities outside of the community, such as college courses, vocational training, fitness activities and recovery support groups. They developed identities related to their vocational development and had a clear sense of their plans beyond River Garden, ‘looking forward to things in the future just having a normal civilised life like a full‐time job, driving a nice car, having your hobbies and your passion and that as well but just getting it all in balance’ (Eric, resident, December 2019).

The five residents who left experienced poor outcomes. For all, relapse either occurred immediately prior or immediately after leaving the community. Two residents experienced and survived near‐fatal overdoses on the same day they left, requiring the use of naloxone and emergency medical treatment. Relapse was often precipitated by becoming unhappy in the community over time, ‘I couldn't do it anymore. And I made a mistake, man, I made a bad choice, and I decided to…go for a drink’ (Brian, ex‐Resident, December 2019). It could also be because of the stress of disclosing leaving, ‘cos I was worried about what to tell my mum…I just…thought, fuck it, I'll just wipe it all out’ (Kyle, ex‐resident, July 2019).

## DISCUSSION

This study theorised the mechanisms through which work‐based residential rehabilitation can support positive outcomes and identified the contextual features that can make positive outcomes more or less likely for different groups. We found that the critical mechanism was instrumental‐motivation and this was experienced by residents with lower problem severity, better health and less socio‐economic disadvantage. Residents with greater contextual barriers may have required greater levels of relational‐motivation. These findings suggest that further solutions may be necessary to support residents with greater contextual barriers.

### Recovery capital as a pre‐requisite for engagement

It has been theorised that residential rehabilitation works by increasing recovery capital, the resources necessary to initiate and sustain recovery [31]. Residential rehabilitation has been framed as particularly suitable for those with lower recovery capital [32] and River Garden itself was designed to increase recovery capital related to vocational skills and employability [21]. However, rather than supporting individuals with low recovery capital, successful engagement in the programme required a high baseline of recovery capital to successfully engage with its demands and challenges. That is, there were limits to the capacity of the programme to operationalise recovery capital and increase the prospects of recovery for those experiencing a deficit of pre‐existing resources [7, 33]. This was mainly because residents with greater contextual resources were better suited to navigate the substantial instrumental and relational challenges. The foundational concept of recovery capital is that individuals from less socio‐economically deprived backgrounds are more likely to experience untreated or ‘natural’ recovery, because of having more resources with which to navigate alternative paths in life [34, 35]. River Garden did not provide active psychosocial interventions, such as group work or individual therapies, which are standard features of residential rehabilitation, because it was designed to replicate the natural environments of working and community life [20, 21]. However, components such as group work can be beneficial for building group cohesion, strengthening peer support, resolving feelings between group members and developing a collective insight into a shared programme of recovery [36]. The omission of psychosocial components from the River Garden design likely contributed to the weak relational mechanisms, hindering the development of cohesive social connections between residents, which ultimately made the programme less suitable for individuals with higher relational support needs. Therefore, it may replicate the outcome inequalities observed under natural conditions.

### The mechanisms of social influence and identity

Social identity models of recovery conceptualise a process where individuals break ties with their existing social groups [37] and develop a recovery‐oriented social identity [8, 38]. This social identity change is framed as the key mechanism of recovery [8]. Residents in River Garden were geographically separated from their previous social groups and entered an abstinent network. However, there were often powerful negative influences between residents and substantial individual or collective resistance to integration. These issues have been observed as part of the challenge of delivering residential rehabilitation elsewhere. For example, studies have found rehabilitation communities polarised by individual differences [39] or engaged in collective resistance to community values [30]. Our findings suggest that social identity change was underpinned by more fundamental relational mechanisms, specifically trust, respect and motivation. In turn, these mechanisms may be inhibited by contextual barriers. This theoretical framing highlights that exposure to abstinent peers alone is not always sufficient to generate motivation. Engagement depends on individual agency and how this has been shaped by contextual factors. Rather than positioning social identity change as the mechanism of recovery, we suggest that social identity change itself is underpinned by deeper relational mechanisms. Understanding these mechanisms is essential to inform the design and implementation of effective interventions.

### Implications and recommendations for policy and practice

Drug policy in Scotland includes an emphasis on expanding national residential rehabilitation capacity as part of the strategy to reduce high rates of DRDs [22]. River Garden was a key beneficiary, along with the Lothian and Edinburgh Abstinence Programme (LEAP), a 3‐month programme involving focused psychosocial intervention, which reports approximately half of its residents sustain abstinence at 4‐year follow up, but a DRD rate commensurate with national rates [40]. Funding priorities included increasing national capacity, improving pathways for vulnerable or complex groups and overcoming barriers to access (including medication, mental health and attitudinal barriers) [41]. Although River Garden could help increase national capacity, there were challenges retaining residents, leaving a population of only four residents by the end of its third year. These challenges related to a limited capacity to manage contextual barriers, including high problem severity, acute health problems and socio‐economic disadvantage. These are the same factors associated with high risk of DRD in Scotland [42, 43]. Furthermore, residents with specific DRD risk indicators (e.g. recent near fatal overdoses) may struggle to navigate the screening and drug testing of prospective residents. For those who did navigate entry, there was evidence that unplanned exit increased overdose risk, consistent with similar residential settings [44, 45, 46]. Therefore, expansion of the programme would not be likely to contribute to aggregate reductions in mortality rates [16, 47], but could continue to support improvements in health and social functioning for suitable residents.

The capacity of this model to support residents with greater contextual barriers could be improved. Implementing the project with a core of functional social enterprises would provide established roles for residents to be assigned to, provide meaningful training and development opportunities and more effectively nurture instrumental motivation. This may improve within River Garden as the instrumental resources are developed over time. Relational mechanisms could be more effectively promoted by integrating a more cohesive therapeutic model into the work‐based programme. This could involve a specific model of recovery (e.g. 12‐step, SMART) and group work therapies to support relational bonds between residents. Ultimately, it is important to find a balance between individualised vocational pathways and collective recovery‐oriented activities to ensure new residents are integrated into a cohesive group characterised by high relational motivation. Finally, staffing resources could be expanded to include more specialist staff, a greater ratio of staff to residents and avoid overreliance on the more informal peer support relationships. Risks around resident intake and exit could be reduced through greater integration with the wider treatment system and formalised pathways to and from other services.

### Limitations

This was a mixed methods process evaluation drawing on specific concepts from realist evaluation. As it did not include experimental methods or quantitative inference, it is not possible to fully differentiate the effects of the contextual resident characteristics from the intervention effect [48]. Findings are based on qualitative analysis and, therefore, reflect participant and researcher interpretations [49]. As such, our theorised mechanisms may not be generalisable to other settings [26]. We have focused on individual‐level context rather than broader policy or governance [50], although infrastructure has been considered in our framework as a programme resource. Although specific concepts from realist evaluation were integrated, we did not attempt to consolidate configurations of context‐mechanism‐outcome. River Garden was a unique work‐based model of residential rehabilitation in its early implementation, so results should be considered as contextually specific. Future research should aim to understand whether different forms of programme resource can promote mechanisms in residents with different contextual barriers, including formal measurement of resident characteristics, comparison to other settings and differences between residents who complete the programme and those who do not.

## CONCLUSION

Residents with higher problem severity, worse physical and mental health and greater socio‐economic disadvantage were less likely to respond to instrumental and relational resources with trust, respect or motivation. The programme best suited residents with high instrumental motivation and least suited residents requiring high relational motivation. Further research should explore how variation in resource implementation affects mechanisms for residents with different contextual facilitators and barriers.

## AUTHOR CONTRIBUTIONS

**Martin Anderson:** Conceptualization; investigation; funding acquisition; writing—original draft; methodology; visualization; writing—review and editing; formal analysis; project administration; data curation. **Lucy Pickering:** Conceptualization; methodology; visualization; writing—review and editing; supervision; formal analysis; funding acquisition. **Mark McCann:** Conceptualization; methodology; visualization; writing—review and editing; supervision; formal analysis; funding acquisition.

## DECLARATION OF INTERESTS

None.

## Supporting information


**Appendix S1.** Participant Observation Proforma

**Appendix S2.** Social Network Interview Topic Guide

**Appendix S3.** Implementer Interview

**Appendix S4.** Table of Mechanisms

## Data Availability

Research data are not shared.
