# Supplementary material for: Context, mechanisms and outcomes of a social enterprise model of residential rehabilitation for problem substance use: A realist‐informed process evaluation
Source: Addiction. 2026 Apr 29;121(9):2414–23. doi: 10.1111/add.70459 (PMC13486912; doi:10.1111/add.70459)
Supplement: Supplementary file 1 [file ADD-121-2414-s001.docx]

# Mixed methods social network data collection procedures

**Study title:** Conducting a process evaluation of a therapeutic community for alcohol and other drugs recovery: social network and identity transitions in a purposively designed social environment

**Introduction**

Obtain written consent to record interview.

Provide a brief overview of the aims of the project. Explain that social networks may influence AOD use and recovery, so we are interested in measuring social networks and discussing them in more detail.

Remind them if they feel uncomfortable to just say so and we can skip the question or topic

**Quantitative scales**

Present participant with the quantitative scales and give them as much time as they need to fill it in. Offer to help complete the questionnaire if they’d prefer not to do it themselves. The scales are the Circumstances, Readiness and Motivation Scales (CRMS) and the Addiction Beliefs Inventory (ABI).

Be clear that these questionnaires are anonymous, and their individual results will not be seen by any intervention staff, and that their answers will not influence their status as a resident.

**Participant background**

Start recording.

Ask participant how old they are, which substances they used, and if they can provide some background information on the circumstances which led to them self-referring to a TC. There is no need to ask for detailed demographic information and drug history as this is captured during their admissions process and will be accessible to the researcher.

**Constructing the ego-net social network map**

Present the participant with the concentric circles template. Explain that this represents their personal social network, with themselves at the centre of the map, and that they will place alters closest to the centre for those they are closest too, and at the outer edge for those they consider distant.

Name generator

Explain that we are going to create a visual representation of their social network. Ask participants to think of *‘people you have had contact with in the past week’*. Ask participant to write the initials of these people on the template, in the appropriate position of closeness. Prompt for alters within the community, visitors, and people they interact with outside the community.

For retrospective maps, the name generator prompt is *‘people you had contact with in the last week before you entered the recovery community’.* Prompt for AOD using peers or others they may have lost contact with after entering the RG admissions process.

If participant names a group or organisation instead of an individual, ask how many people are these, how many did they interact with, what type of activities take place (what, when, where, who, how).

Names are to be written on sticky notes then placed on the concentric circles. Ask participant to consider how the alters are placed in comparison to each other, and move them around if necessary.

Name interpreter

Explain that we are going to capture additional details about the people in the social network. Ask participant to draw a green circle around alters who had a positive influence on AOD recovery or healthy lifestyle, and a red circle around alters who were a negative influence. Use the name interpreter cards to collect additional information about each alter: age, gender, alcohol use, other drug use, if they use treatment services, if they are in recovery, and what is their relationship to the participant.

Alter-alter ties

Explain that we are going to capture details on how connected the people in the network are to each other. Since this is a residential environment, it can be assumed that all participant will know each other quite well. Alter-alter ties will be elicited based on alters who the participant considers having *‘a close relationship’.* A close relationship would be defined as one where the alters discuss important matters with each other and spend their free time/leisure time together. Ask participants to draw a black line between all alters who have close relationships.

For a lesser-weighted measure of alter-alter ties, also ask who in the network simply knows each other well enough to chat. We will then have three levels: closely connected, connected, unconnected.

At this stage the network map will visualise the degree, closeness, and positive/negative influence of alters, and alter-alter ties.

**Prompts for qualitative interview**

The construction of the network map will act as a prompt for a qualitative interview. When participants are drawing the map, ask them to explain why they are placing alters in certain positions, why they have marked alters as positive or negative, the nature of their relationship with the alters and the alters relationships with each other. Specifically focus on these relationships in reference to their influence on AOD use and recovery. Many of these topics will occur naturally during the drawing of the network maps. They can be prompted if they don’t occur naturally. Some questions could include:

Social support

- ‘can you explain how [alter] has influenced your recovery?’
- ‘who would you be most likely to ask for support or advice?’
- ‘if you had a problem who would you approach first?’
- ‘how does the support you receive here compare to other forms of support (e.g. professional intervention)?’

Nature of relationships

- ‘what sort of relationship do you have with [alter]?’
- ‘can you explain what makes [alter1] a closer relationship than [alter2]?’
- ‘what made this relationship positive/negative?’
- ‘have you experienced any conflict or observe conflict between others?’

Social influence

- ‘how did these relationships influence your decision to pursue recovery?’
- ‘how does a network of abstinent/recovery peers influence your wellbeing?’

Network structure

- ‘who in the network meets in smaller groups and what sort of activities are involved’ (what context/activities)? (cliques)
- ‘who in the network can you get difference sources of information and advice from? (closure)
- ‘which different activities and communication can you have with different groups’ (opportunity/constraint)
- ‘can you explain why [two unconnected alters] are not close?’ (structural holes)

**Additional prompts for retrospective map**

Social support

- ‘can you explain how [alters] supported you during your AOD use’
- ‘who (if any) influenced your decision to pursue recovery’
- ‘if you had a problem who would you have approached first’
- ‘how did this support compare to support you are receiving now’

Nature of relationships

- ‘What sort of relationship did you have with [alter]
- ‘What makes [alter1] a close relationship than [alter2]
- ‘What made this relationship positive or negative’
- ‘did you experience any conflict or observe conflict between others’

Social influence

- ‘how did these relationships influence your AOD use’
- ‘how did these relationships influence your decision to pursue recovery?’
- ‘how did a network of AOD-using peers influence your wellbeing?’

Network structure

- ‘who in the network met in smaller groups and what sort of activities were involved’ (what context/activities)? (cliques)
- ‘who in the network could you get difference sources of information and advice from? (closure)
- ‘which different activities and communication could you have with different groups’ (opportunity/constraint)
- ‘can you explain why [two unconnected alters] were not close?’ (structural holes)

**Conclusion**

Thank participant for taking part in the interview and ask if they have any questions or anything else they want to talk about. Switch off recording device and store all data at SPHSU.
