# Supplementary material for: Context, mechanisms and outcomes of a social enterprise model of residential rehabilitation for problem substance use: A realist‐informed process evaluation
Source: Addiction. 2026 Apr 29;121(9):2414–23. doi: 10.1111/add.70459 (PMC13486912; doi:10.1111/add.70459)
Supplement: Supplementary file 2 [file ADD-121-2414-s002.docx]

**Topic guide for qualitative interviews with programme implementers**

**Introduction**

Thank you for taking part in this interview. As you know, I have been conducting fieldwork with residents of River Garden. The purpose of this interview is to help me check some of the theories I have developed during this fieldwork. These theories are concerned with what aspects of the programme work, for whom, under what circumstances. You are being interviewed because your role in developing or implementing *River Garden* means you will have insights into how it works (or doesn’t), the importance of contextual factors, and the likely outcomes of the programme.

| Question | Logic |
| --- | --- |
| I understand you have been closely involved with the implementation of *River Garden*. From your perspective, what are the things that are most important for me to understand about how the programme works? | Introductory question demonstrates that the programme architecture is the focus of the interview. |
| I am interested in how the San Patrignano, Basta, and Delancey Street models are adapted for Scotland. What have been the most important changes made to the programme?  *What issues made these changes necessary? (E.g. a reactive response to circumstances, or a proactive, conscious decision).*  *How did these changes come about? Who initiated them?* | Looking for transferability 1 (programme adaptions)  In a time-sensitive model, these programme-level outcomes become the new context for the next programme phase. |
| Which kind of people are more likely to benefit from River Garden than others?  *Is resident background important? If so, what is it about background that influences what works?*  *(Potential prompts: trauma, family, severity, age, class, crime.)*  *Has it been necessary to change the programme to make it more effective for a broader range of people?*  *What would make someone unsuitable to become a resident?* | Exploring context 1 (resident characteristics) |
| What has changed in how well RG was working (or not) in the early stages compared to now?  *What was working well?*  *What wasn’t working well?*  *How are resident experiences influenced by the types of work and training available?*  *Has this changed from the early stages to now?*  *What are the experiences of new residents compared to more experienced residents?*  *Has this changed from the earlier stages to the later stages?*  *What about the balance between the number of new and experienced residents?*  *How has the infrastructure developed since the early stages?* | Exploring context 2 (environmental characteristics) |
| How has the *River Garden* culture developed since the early stages?  *What are the main features of the River Garden culture and how do these aspects of the culture help recovery?* | Exploring context 4 (community culture) |
| I have seen that residents have a lot of freedom to interact with people outside of the programme. What are your thoughts on how much freedom residents should have?  *What is it about the level of freedom that works or doesn’t work?*  *In what ways does interaction with external organisations (twelve-step, NHS services) influence residents?*  *Have there been any adaptions to this aspect of the programme?*  *How has COVID-19 affected this?* | Exploring context 3 (characteristics of the design/transferability) |
| To what extent is the programme, and residents, integrated with regional and national recovery movement?  *Are there positives for residents engaging with the recovery movement? And are there any negatives?*  *Are there any reasons for residents not engaging?*  *Are residents more or less integrated with recovery than River Garden as an organisation?*  *What about other national movements, e.g. #StopTheDeaths, Drugs Research Network Scotland, Harm Reduction?* | Exploring context 4 (policy environment) |
| How close has the operational management of *River Garden* been to board policy and direction?  *Has this changed since the programme started?*  *In what ways has it changed and why?*  *How does this affect how the programme is delivered?*  *What are the pros and cons of board involvement in the programme operation?*  *Does this lead to the programme deviating from the original board vision? If so, in what ways?* | Exploring context 5 (IFDAS board) |
| What role does peer support play compared to other forms of help, particularly within *River Garden*?  *How does it compare to peer support available in twelve-step groups, recovery cafes etc?*  *What factors are important in making sure people respond well to peer support?*  *Can you provide an example of a time when peer support has worked and one when it has not worked? Why was this?*  *Any adaptions to make peer support more effective?* | Looking for mechanisms 1(peer support) |
| What is it about providing people with purposeful work activity that helps them recover?  *It seems like some residents will respond more positively to structured work than others. What factors would explain that?*  *Has it been necessary to adapt anything relating to the work routines?* | Looking for mechanisms 2 (structured purposeful activity) |
| How do you think the rules and supervision arrangements help residents recover?  *What are the most important rules?*  *Have residents been invested in the rules or has there been resistance to some rules?*  *How do you ensure residents are invested in the rules?*  *Have the rules been adapted?*  *What are the consequences of rule breaking for individual residents and the programme as a whole?*  *(Potential prompts: phones, smoking, how the level of freedom from supervision affects rule adherence).* | Looking for mechanisms 3 (rules and supervision) |
| It seems that removing people from their previous environment is an essential part of the programme. What is it about offering a residential programme that is important?  *What is the most important benefit for residents of being offered long-term (three years) accommodation?*  *What sort of factors could lead to somebody becoming unhappy with the environment?*  *What are the implications when new residents come in with potentially challenging behaviours and issues?*  *Could this be avoided? If so, how? (Potential prompt: careful recruitment?)* | Looking for mechanisms 4 (abstinent environment) |
| There is a theory that forming new connections is an important aspect of recovery. What are the important factors in ensuring that residents form positive connections?  *I have noticed that residents who work together seem to form closer connections. What are the implications of this?*  *Have any residents become disconnected or isolated??*  *What are the consequences of resident conflict and how is this managed?* | Exploring outcomes 1 (connection) |
| I have seen some residents achieve long-term abstinence. Others have left the programme and relapsed. What are your opinions about what happened with those who have left?  *Why do some people leave and other stay?*  *Are there any important changes that could be made to the programme to reduce these occurrences?*  *The possibility of relapse and overdose is a recognised risk of abstinence-based programmes, how is this managed?*  *How appropriate is it to remove somebody from the programme if they relapse?* | Exploring outcomes 2 (relapse, leaving) |
| It seems that a lot of work is involved in managing resident health. What is it about the programme that helps someone improve their physical and mental health?  *Could the pressures of the programme lead to worse health in some circumstances? (E.g. providing peer support may be stressful).*  *Examples of how health is managed?* | Exploring outcomes 3 (physical and mental health) |
| 1. Why are trust and respect so important for residents? I have noticed these are key issues in how they respond to peer support. 2. How important is it to show gratitude for the support they are being given? 3. What are the consequences if residents become unmotivated? 4. What happens if residents feel that there is a lack of reciprocation when it comes to gratitude and respect? 5. Why is the level of freedom to interact outside the programme beneficial to some and destabilising to others? 6. Some difficulties in the early stages seemed to result from the fact the infrastructure was still being developed. What was it about this that caused problems? 7. What is it about developing a robust community culture that helps the programme remain stable? | Exploring mechanisms (trust, respect, safety, gratitude, motivation etc. Any other mechanisms introduced by the interviewee can be addressed here too.) |
| My study is interested in the ways River Garden is adapted to work in the Scottish context. Would you agree with these changes that I have noticed?   - *Relaxing of smoking policy* - *Reduced freedom for new residents to pursue external support (e.g. twelve-step).* - *Less integration with wider recovery movement.* - *More emphasis on avoiding external relationships.* - *Reduced board oversight in daily operations.* - *Development of cohesive culture (e.g. emphasis on gratitude, surrender).* - *Limiting resident numbers and focusing on building infrastructure.* - *More selective about residents with greater problem severity* - *Speeding up of entry procedures.* - *Greater fluidity in programme stages (e.g. not automatically progressing someone to peer worker at 12 months).*   *What other adaptions may be necessary?* | Exploring transferability (validating programme changes observed). |

**Thank you for taking part in this interview**.
