# Supplementary material for: Context, mechanisms and outcomes of a social enterprise model of residential rehabilitation for problem substance use: A realist‐informed process evaluation
Source: Addiction. 2026 Apr 29;121(9):2414–23. doi: 10.1111/add.70459 (PMC13486912; doi:10.1111/add.70459)
Supplement: Supplementary file 3 [file ADD-121-2414-s003.docx]

Appendix D – Table of Mechanisms

| **Mechanism** | **Definition** | **Presence** | **Absence** |
| --- | --- | --- | --- |
| Relational-trust | The ability to trust people in the community on an interpersonal level | *“I know I can have a laugh with them and … be myself around them … I feel most comfortable and connected with these ones and that’s probably who I trust the most as well” (Ian, Resident, May 2019).* | *““It is difficult to trust people in here … when I retract to my own environment or room, I question closeness of people in here” (Stuart, Resident, Feb 2020).* |
| Relational-respect | The ability to respect people in the community on an interpersonal level | ‘People [who] are pretty sound … tend to stick together (Ian, Resident, May 2019) | *“If you spoke to somebody in a normal way, they would think you were weird … it was just all flinging pelters at each other, all giving abuse” (Kyle, Ex-Resident, July 2019).* |
| Relational-motivation | Whether a resident is motivated to form close relationships with other residents or the wider recovery community | *‘It was a good time, during the summer, after everything kind of settled down again … it definitely made the people that were still here a stronger community’ (Ian, Resident, Dec 2019)* | *“I couldn’t go back into that accommodation block, now, with the boys … sharing bathrooms again, and sitting round at mealtimes with everybody … I don't want to do that again … I've had enough” (Brian, Ex-Resident, Dec 2019).* |
| Instrumental-trust | The extent to which residents trust that following the structures and routines are of benefit to themselves rather than the project | *“Not having that purpose drags you down … Whereas here okay, fair enough, I’m an alcoholic but I’m also just doing all this stuff, work … you value yourself again, because I’m a really productive person, I’ve got a lot to give to society, just had a shit couple of years.” (Paul, Resident, August 2019).* | *‘I’ve always tree planted … and you’re always getting paid for it … you’re overworked in here. Sometimes you feel like you’re in the … slaves or something like that’ (Kyle, Resident, April 2019).* |
| Instrumental-respect | Whether residents respect the structures and routines of the community | *““I’ve got … tickets [qualifications] for outdoor work for like the trimmers and brush cutters … motor training for the tractors. I’m also doing really well at college … looking forward to things in the future just having a normal civilised life” (Eric, Resident, Dec 2019).* | *We need to be strict with, you know, you get up in the morning, you go to breakfast, you do this, you work, you don’t have your phone, you don’t smuggle phones in, you use your phone not on work times, you honour your commitments, if you’re on cooking rota you don’t just skip it and go outside … at some point you need to sit people down and say, ‘this is the structure River Garden has set, we expect you to follow the rules’, like, no gambling, … no smoking indoors, no threats, no, you know, you’re here to do your job.” (Nigel, Staff, August 2019)* |
| Instrumental-motivation | The level of motivation towards routine work and vocational training opportunities | *“I’ve now got a social enterprise that I kind of have started … so I’ve got my kind of area of focus, as well as all the other day to day stuff … I think because I’ve worked hard and tried to be dependable, reliable and like trustworthy, that I am relied upon by the staff as well” (Paul, Resident, Feb 2020)* | *“They were impressed by my work ethic at first … you just burn out after a wee while … you lose the will” (Kyle, Resident, April 2019).* |
