# Supplementary material for: Context, mechanisms and outcomes of a social enterprise model of residential rehabilitation for problem substance use: A realist‐informed process evaluation
Source: Addiction. 2026 Apr 29;121(9):2414–23. doi: 10.1111/add.70459 (PMC13486912; doi:10.1111/add.70459)
Supplement: Supplementary file 4 [file ADD-121-2414-s004.docx]

 **Participant observation proforma**

| **Date** | **Start time** | **End time** |
| --- | --- | --- |
|  |  |  |
| **Locations visited** | **Participants** | **Keywords** |
|  |  |  |

Provide a detailed account of where you were, who you were with, and what activities you were doing. Pay particular attention to:

- Any talk about AOD use and recovery, however brief
- Any mentoring observed, participated in or discussed by participants
- Any discussion of the intervention strategy and delivery, either positive or negative (e.g. comments on the structured work routines, the influence of peer support)
- Relations of support (which peers are offering support to others in recovery, which form does it take, is it emotional or practical support)
- Any talk about personal change/transition/growth since entering the community (sense of self, identity, habits and routines)
- Any observations about the structure of social networks in the community (e.g. is everyone close, are there cliques, does individual behaviour appear constrained by networks?)

Participant-observation in Rapid Ethnographic Appraisal involves **active** **data collection**. Watch and listen to see how AOD recovery is discussed spontaneously, but when that happens, it may be appropriate to asking probing questions (can you tell me a bit more about that, why do you think that might be the case etc).

Ensure your field notes contain your reflections on the day’s data collection. These will include:

- How you felt, and what emotions were triggered by your encounters and observations (this is important as your field notes are necessarily subjective, so reflections on your emotional state allow the team to take that into account during analysis)
- Any new areas for enquiry that emerged, and how you might actively engage participants in relation to them in future
- Any themes you feel are emerging, and how they might fit into end-of-data-collection analysis
